# Combined surgery and radiation improves survival of tonsil squamous cell cancers

**DOI:** 10.18632/oncotarget.20122

**Published:** 2017-08-10

**Authors:** Anurag K. Singh, Christina Mimikos, Adrienne Groman, Shiva Dibaj, Alexis J. Platek, David M. Cohan, Wesley L. Hicks, Vishal Gupta, Hassan Arshad, Moni A. Kuriakose, Graham W. Warren, Mary E. Platek

**Affiliations:** ^1^ Department of Radiation Medicine, Roswell Park Cancer Institute, Buffalo, NY, USA; ^2^ Department of Head and Neck/Plastic and Reconstructive Surgery, Roswell Park Cancer Institute, Buffalo, NY, USA; ^3^ Department of Biostatistics, Roswell Park Cancer Institute, Roswell Park Cancer Institute, Buffalo, NY, USA; ^4^ Department of Radiation Oncology, Medical University of South Carolina, Charleston, SC, USA; ^5^ Department of Cell and Molecular Pharmacology, Medical University of South Carolina, Charleston, SC, USA; ^6^ Department of Health, Nutrition and Dietetics, Buffalo State College (SUNY), Buffalo, NY, USA

**Keywords:** tonsillar fossa, TORS, SCCHN, HPV, IMRT

## Abstract

**Objective:**

The study evaluated the addition of surgery (S) to radiation (RT) on survival of squamous cell carcinomas (SCC) of tonsillar-fossa (TF) in a modern cohort with similar epidemiology and treatment as current patients.

**Study Design:**

Retrospective analysis utilizing Surveillance, Epidemiology, and End Results (SEER) Program data.

**Results:**

For all stages combined TF patients who received S+RT had superior OS (*p* < 0.01) and DSS (*p* < 0.01). For each stage OS and DSS was superior for S+RT (*p* < 0.05). In multivariate analysis, HRs for OS were statistically significantly higher for TF patients (stage 2, 3, and 4) receiving RT alone (*p* < 0.001).

**Materials and Methods:**

TF SCC patients treated with either S+RT or RT alone between 2004 and 2011 were examined (*n* = 6,476). Primary outcome measures included overall survival (OS) and disease specific survival (DSS). Cox proportional hazard ratios (HR) were estimated for patients treated with S+RT compared to RT alone.

**Conclusions:**

OS and DSS were superior for all stages combined and for stages 2, 3, and 4 in TF patients who received S+RT compared to RT alone.

## INTRODUCTION

Squamous cell cancer of the head and neck (SCCHN) occurs annually in over 550,000 people worldwide [1, 2]. In the US, it occurs in 59,340 people per year and causes 12,290 deaths annually [3]. Tobacco use has been the major risk factor for developing SCC of the head and neck [4]. In recent decades, a higher rate of human papilloma virus (HPV) associated SCCHN has been reported, especially in tumors arising in the oropharynx [5, 6]. Presence of HPV in tumor specimens has been associated with improved survival [7, 8–13].

During this period of shifting epidemiology, advances in treatment including the use of concurrent chemotherapy and radiation therapy (RT) [14, 10] as well as the development of advanced radiation techniques such as intensity modulated radiation therapy (IMRT) have improved survival [15]. Recent surgical advances such as trans-oral robotic surgery (TORS) have been reported to be feasible without sacrificing outcomes in oropharyngeal SCC [16, 17]. Surgical approaches have dual advantages of significant tumor debulking and providing enhanced staging information. Theoretically, these advantages may produce a survival benefit. For early stage tonsil SCC, one analysis of the SEER database has shown improved survival with tonsillectomy added to radiation therapy [18].

We recently compared survival outcomes between base of tongue and tonsillar fossa patients in a modern cohort within SEER [19]. By virtue of the sheer size of the database and modernity of this cohort, this analysis is likely to have similar incidence of human papilloma virus (HPV) associated tumors and similar frequency of intensity modulated radiation therapy and chemotherapy as current practice, though these viral and treatment variables are not specifically captured in the SEER database. There have been recent/ongoing clinical trials (i.e., Radiation Therapy Oncology Group 1221/NCT01953952 and Eastern Cooperative Group 331/NCT01898494) that attempted TORS in both HPV positive and HPV negative tonsil cancer. Pending results of these ongoing trials, this examination sought to evaluate the effect of the addition of surgery to RT (S+RT) on survival of all stages of squamous cell carcinomas (SCC) of the tonsillar-fossa (TF) in this SEER cohort.

## RESULTS

The cohort included 6,476 primary TF SCC patients who were treated with surgery with adjuvant radiation or with radiation alone between 2004 and 2011. The demographic, clinical, and pathologic characteristics of the TF cohort by treatment are displayed in Table 1. TF patients who received surgery with radiation were significantly younger than those treated with primary radiation (median age 55 vs 57, *p* < 0.001) and had a greater percentage of T1 tumors (41.0% vs 15.8%, *p* < 0.001). The cohort is predominantly white with similar distribution of gender between treatment groups.

**Table 1:** Descriptive statistics of tonsil patients by treatment (*n* = 6476)

*n* (%) or Median (Range)	Surgery with Adjuvant Radiation (*n* = 3195)	Radiation only (*n* = 3281)	*P*-value
Age	55 (25, 87)	57 (24, 92)	< 0.001
Age Group ≤ 50 51–60 61–70 71–80 > 80	984 (30.8%) 1387 (43.4%) 643 (20.1%) 164 (5.1%) 17 (0.5%)	685 (20.9%) 1400 (42.7%) 808 (24.6%) 303 (9.2%) 85 (2.6%)	< 0.001
Sex Male Female	2686 (84.1%) 509 (15.9%)	2767 (84.3%) 514 (15.7%)	0.770
Race White Black Other	2883 (90.2%) 194 (6.1%) 118 (3.7%)	2787 (84.9%) 363 (11.1%) 131 (4.0%)	< 0.001
N Stage N0 N1 N2 N3	520 (16.3%) 831 (26.0%) 1720 (53.8%) 124 (3.9%)	600 (18.3%) 750 (22.9%) 1761 (53.7%) 170 (5.2%)	< 0.001
T Stage T1 T2 T3 T4	1311 (41.0%) 1332 (41.7%) 251 (7.9%) 301 (9.4%)	519 (15.8%) 1593 (48.6%) 590 (18.0%) 579 (17.6%)	< 0.001
Stage I II III IV	157 (4.9%) 253 (7.9%) 810 (25.4%) 1975 (61.8%)	89 (2.7%) 311 (9.5%) 750 (22.9%) 2131 (64.9%)	< 0.001
Grade (*N* = 5336) I II III IV	91 (3.1%) 1174 (40.4%) 1585 (54.6%) 54 (1.9%)	102 (4.2%) 1082 (44.5%) 1208 (49.7%) 40 (1.6%)	< 0.001
Year 2004 2005 2006 2007 2008 2009 2010 2011	345 (10.8%) 356 (11.1%) 326 (10.2%) 361 (11.3%) 403 (12.6%) 449 (14.1%) 461 (14.4%) 494 (15.5%)	321 (9.8%) 313 (9.5%) 341 (10.4%) 360 (11.0%) 375 (11.4%) 524 (16.0%) 521 (15.9%) 526 (16.0%)	0.046

Associations between columns were statistically assessed using the Wilcoxon Rank Sum test (or Kruskal-Wallis test) in the case of ordinal responses and the Pearson Chi-square test for categorical responses.

**Figure 1:**
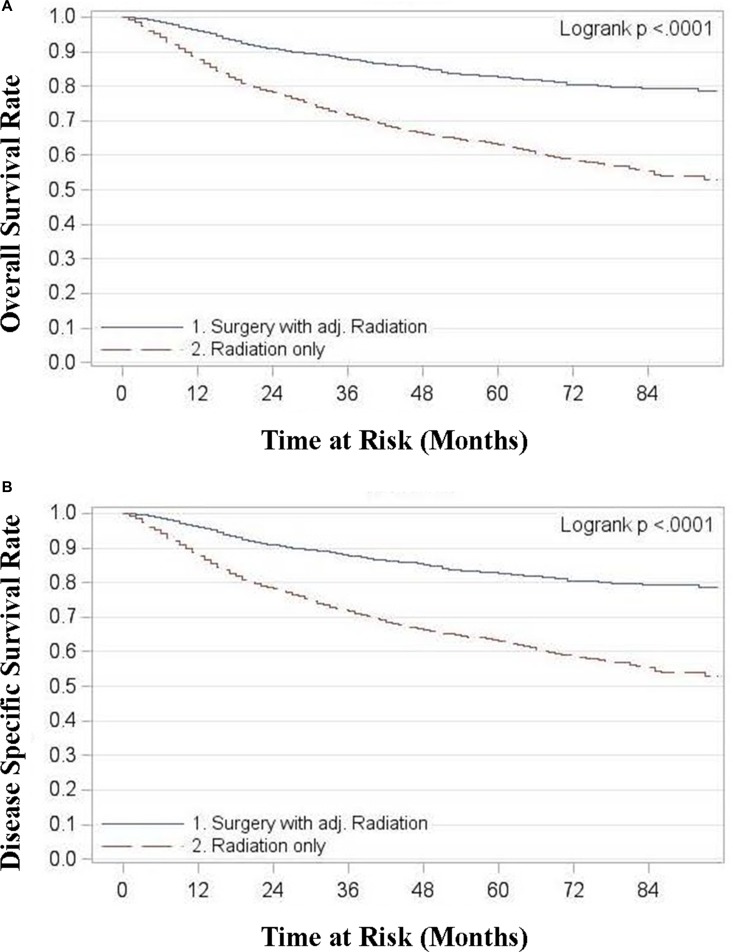
**(A)** Overall survival in tonsillar fossa patients treated with surgery and adjuvant radiation vs. radiation only, all stages **(B)** Disease specific survival in tonsillar fossa patients treated with surgery and adjuvant radiation vs. radiation only, all stages.

**Figure 2:**
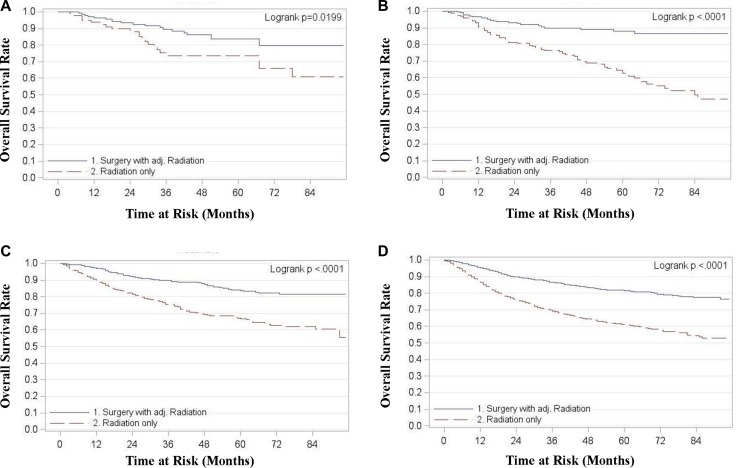
Overall survival in tonsillar fossa patients treated with surgery and adjuvant radiation vs. radiation only **(A)** Stage 1 **(B)** Stage 2 **(C)** Stage 3 **(D)** Stage 4.

**Figure 3:**
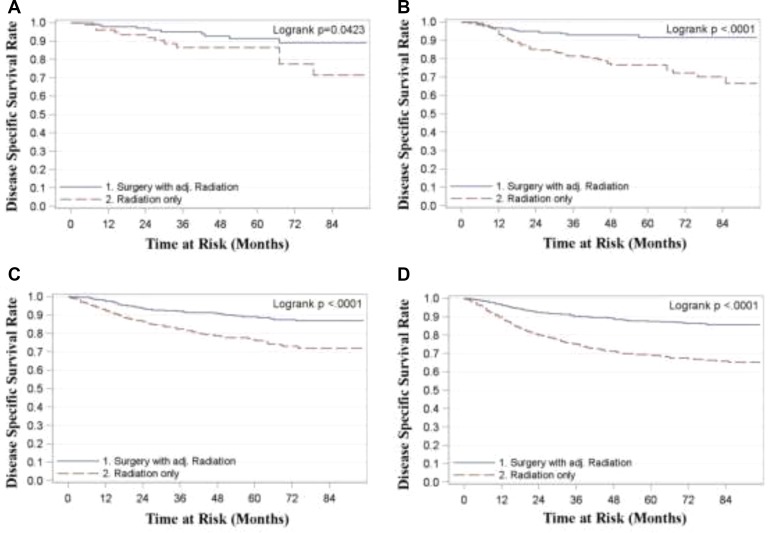
Disease specific survival in tonsillar fossa patients treated with surgery and adjuvant radiation vs. radiation only **(A)** Stage 1 **(B)** Stage 2 **(C)** Stage 3 **(D)** Stage 4.

**Figure 4:**
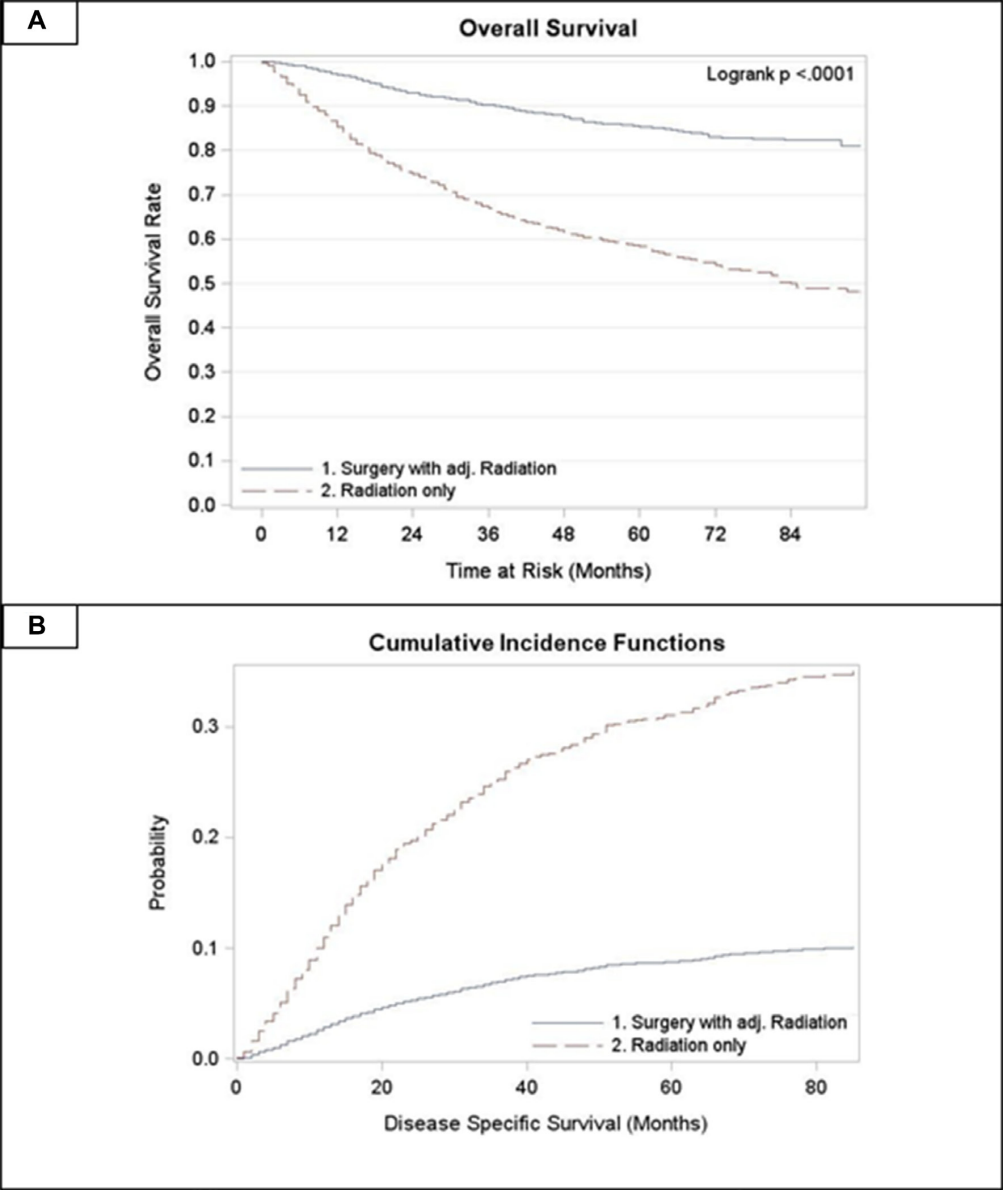
Propensity score weighted curves **(A)** Overall survival in tonsillar fossa patients treated with surgery and adjuvant radiation vs. radiation only **(B)** CIF for disease specific survival in tonsillar fossa patients treated with surgery and adjuvant radiation vs. radiation only.

For all stages combined, TF patients who received surgery with radiation had significantly superior OS and DSS compared to patients treated with radiation alone (*p* < 0.0001) (Figure 1). Five year OS rates for surgery with adjuvant radiation (S+RT) vs. radiation alone (RT) for all stages combined were 0.83 (0.81, 0.85) and 0.63 (0.61, 0.66) respectively. Five year DSS rates for all stages combined were 0.89 (0.87, 0.9) for S+RT and 0.72 (0.70, 0.74) for RT alone. When stratified by stage TF patients treated with S+RT had significantly superior OS and DSS compared to those treated by RT for each stage (*p* < 0.05) (Figures 2 and 3). The propensity score weighted KM plot for OS and propensity score weighted CIF curve for DSS support these findings (Figure 4).

In multivariate analysis, the Hazard Ratio (HR) was estimated for OS comparing S+RT vs. RT for all stages combined and then stratified by stage (Table 2). Models were adjusted for age as a continuous covariate, gender, race, *T* and *N* stage individually, and tumor grade. HRs were significantly higher for TF patients who received RT alone compared to those who received S+RT for all stages combined and for stages 2, 3, and 4 (*p* < 0.001). The estimate from the propensity score weighted hazard ratio model for OS remained significant (HR: 3.74, *p* < 0.001).

**Table 2:** Cox proportional hazard models of tonsil patients by treatment for overall survival (2004–2011)

	Univariate Model		^a^Multivariate Model	
	^b^HR (95% CI)	^c^*p*-value	^b^HR (95% CI)	^c^*p*-value
**All Stages** Surgery with Radiation Radiation Only	1.00 2.59 (2.30, 2.92)	< 0.001	1.00 1.92 (1.70, 2.18)	< 0.001
**Stage 1** Surgery with Radiation Radiation Only	1.00 2.04 (1.11, 3.77)	0.023	1.00 1.61 (0.85, 3.08)	0.147
**Stage 2** Surgery with Radiation Radiation Only	1.00 3.50 (2.21, 5.54)	< 0.001	1.00 3.01 (1.88, 4.84)	< 0.001
**Stage 3** Surgery with Radiation Radiation Only	1.00 2.55 (1.98, 3.29)	< 0.001	1.00 2.16 (1.67, 2.81)	< 0.001
**Stage 4** Surgery with Radiation Radiation Only	1.00 2.53 (2.18, 2.92)	< 0.001	1.00 2.16 (1.85, 2.51)	< 0.001

^a^Adjusted for continuous age, gender, race, *T* stage, *N* Stage and grade.

^b^Hazard ratio, 95% confidence limits and *p* values associated with a Cox Proportional Hazards model.

^c^Associations considered statistically significant at a *p* value < 0.05.

## DISCUSSION

This analysis found significantly improved OS and DSS for all stages (I–IV) TF SCC with surgery and radiation compared to radiation alone. On multivariate analysis adjusting for age, gender, race, T stage, N stage, and tumor grade OS remained statistically significantly superior for stages II, III, and IV (*p* < 0.001).

The absence of OS benefit in stage I patients in this analysis is consistent with the meta-analysis findings reported by Morisod et al. showing equivalent OS of 90% in early stage TF patients treated with either radiation or trans-oral surgery [17]. However, an earlier SEER review by Holliday et al. covering 1988 to 2006 found that patients with stage I and II TF SCC had a higher 5 year OS and DSS with tonsillectomy followed by adjuvant radiation therapy compared to patients receiving radiation therapy alone (83% and 90% versus 64% and 76%) [18]. The long time frame, 1988 to 2006, of the Holiday et al. analysis is a weakness due to the tremendous differences in epidemiological factors (such as incidence of smoking and HPV) and significant evolution of therapy (from the era of two-dimensional therapy in the late 1980s through to the advent of intensity modulated radiation therapy in the 2000s and the utilization of chemotherapy in the later period.) The present analysis, by restricting the period studied to the modern period of 2004–2011, limits these weaknesses.

### Benefits of primary surgery in TF SCC

Surgery produces significant tumor debulking and provides enhanced staging information. Theoretically, these advantages may produce a survival benefit by reducing tumor burden and also by stage migration. In well selected cases, some patients may avoid chemotherapy and/or radiation and thus be free from the toxicities that accompany chemotherapy and radiation. There is an inherent selection bias for lower stage patients to be offered surgery, due to the technical ease of resecting smaller tumors and the potential to spare additional side effects of radiation and/or chemotherapy [20, 21] Surgical resection changes staging up to 40% of the time, with approximately 24% of patients getting down-staged [20, 21].

Using data from a multi-institutional cooperative group trial, Ang et al., demonstrated that both HPV status and smoking exposure impact survival with the best survival in HPV positive non-smokers and the worst in HPV negative patients with a greater than 10 pack year history [7].

With the caveat that this data suffers from selection bias, some evidence suggests that primary surgical therapy with appropriate adjuvant treatment may be a reasonable method to intensify treatment in poor prognosis patients. A multi-institutional review of patients who underwent Transoral Robotic Surgery (TORS) reported good 2 year OS of 91% and DSS of 94%, in a cohort of predominantly oropharyngeal cancer patients [22]. Interestingly, this study did not find a significant difference in survival between HPV + vs HPV – patients [22]. This finding was supported by Cohen et al., who observed disease control with primary surgical intervention for both HPV + and HPV – OPSCC at two year follow up, with OS and DSS 80.6% and 92.6% respectively [23]. Results of a systematic review conducted by Wang et al yielded substantially improved outcomes for HPV – OPSCC patients treated with primary surgery when compared to OPSCC patients treated with radiation [24].

In contrast, Kelly et al. reviewed the National Cancer Database and found no change in survival outcomes among 1044 patients with newly diagnosed cT1–2 N1–2b HPV-negative OPSCC when treated with primary surgical resection vs chemoradiation. Interestingly, 59% of primary surgery patients required adjuvant chemoradiation leading the authors to conclude that “further research should focus on better selection of surgical patients who are less likely to require adjuvant” chemoradiation. [25] We completely agree with this sentiment and note that 41% of the surgical patients described by Kelly et al. who did not require adjuvant chemoradiation therapy and achieved equivalent survival effectively achieved treatment de-intensification by selecting surgery first. Moreover, the adjuvant radiation dose (often 60 Gray (Gy)) is considerably lower than the dose used for radiation alone or chemoradiation (usually 70 Gy,) arguing that addition of surgery (if done with limited toxicity) does indeed allow for a de-intensification of both chemotherapy (fewer cycles) and radiation (less dose).

### Weaknesses of the SEER analysis

Detailed pathological factors not documented in SEER, such as extracapsular extension, may have confounded our analyses. Other factors not accounted for in the SEER database including HPV status, the use of chemotherapy, type of radiation, and patient functional qualities and comorbidities may have also confounded analyses.

Results from investigations of surgical patients may be influenced by selection bias as these patients have fewer comorbidities, younger age, lower stage disease, or less bulky disease than their stage-matched peers who undergo non-operative management only. Additionally, there is no clear method to determine which surgical procedures were performed for salvage in the SEER population.

### Future directions

This analysis shows improved OS in SCC TF patients treated with surgery and adjuvant radiation. There is already evidence to support lower cost with primary surgical therapy, with or without radiation [26]. Further, innovations such as trans-oral surgical intervention, including TORS and Transoral Laser Microsurgery (TLM), in early stage disease shows substantially improved quality of life [22, 27]. Both techniques offer improved cosmetic and functional outcomes over traditional open approaches.

Depending on the results of the recent and ongoing prospective studies, if surgery can truly be done with minimal toxicity, then radiation and chemotherapy may be significantly de-intensified. If the phase 2 randomized study, NRG Oncology HN002 (NCT02254278) suggests that definitive chemoradiation doses can be safely reduced, then it might open the possibility of doing surgery and even further chemoradiation dose de-escalation.

## MATERIALS AND METHODS

We queried the Surveillance, Epidemiology and End Results (SEER) program data of the National Cancer Institute between 2004 and 2011. We selected TF ICD-O-3 head and neck cancer location codes as follows: C090, C091, C098 and C099. Histologic codes applied for squamous cell carcinoma were 8050–8084. Patients treated by primary radiation and surgery with adjuvant radiation were included. Patients with distant metastases were excluded. The cohort included 6,476 patients. The American Joint Committee on Cancer (AJCC) Seventh edition guidelines were applied to the T (all codes not in 99, 00, and 05) and N (all codes not in 99) SEER stages of the tumors to determine the overall stage of each tumor.

The SEER data is the most comprehensive population-based data source of cancer incidence and survival in the United States, and covers approximately 28% of the US population [28, 29]. This data set provides information on patient demographics, cancer site, histo-morphologic classification, clinical stage at diagnosis, treatment history and sequence (surgery and/or radiation), follow-up duration and vital status [28, 29]. SEER does not, however, provide information on smoking history, chemotherapy use, type of radiation (conventional vs. IMRT), HPV status, or detailed data on disease recurrence.

### Statistical methods

Overall survival (OS), the primary endpoint, was defined as the time (in months) from diagnosis to death from any cause. Patients alive at the date of last follow-up were censored. Disease-specific survival (DSS) was defined as time (in months) from diagnosis to death specifically from cancer. Patients dying from other causes were censored at date of death and those alive were censored at the date of last follow up. Age as a continuous covariate, race, gender, tumor grade, *T* stage, *N* stage, and overall stage were considered as possible confounding factors. Associations between these variables and treatment (radiation alone or surgery with adjuvant radiation) were analyzed using the Pearson Chi-square test for categorical variables and Wilcoxon Rank Sum test for continuous variables. Multivariate proportional hazards modeling results were used to assess the adjusted effect of treatment on overall and disease-specific survival. Relative prognosis was summarized using estimates and 95% confidence limits for the hazard ratio (HR). Unadjusted differences in overall and disease-specific survival by treatment are shown using Kaplan-Meier methods for all stages combined and by stage.

To account for potential confounding covariates a propensity score weighted model was used to examine the treatment differences after accounting for age, race, gender, tumor grade, N stage and T stage. Weights were obtained from a logistic regression model and were used to weight the observations in the proportional hazards model. A competing risk cumulative incidence function (CIF) was used to examine the effect of treatment on the disease specific survival. Propensity weights were used to account for potential confounding factors. All associations were considered statistically significant at an alpha error < 0.05 (*P* value 0.05). All analyses were performed using SAS version 9.4.

## CONCLUSIONS

In this SEER cohort, OS and DSS were superior for all stages in TF patients who received surgery with adjuvant radiation compared to those who received radiation alone. Multivariate analysis showed this OS advantage was retained in all but stage 1 patients. A prospective study examining surgery plus adjuvant radiation versus primary radiation should be considered.
